# The impact of medial temporal and parietal atrophy on cognitive function in dementia

**DOI:** 10.1038/s41598-024-56023-3

**Published:** 2024-03-04

**Authors:** Noor Alia Susianti, Astuti Prodjohardjono, Amelia Nur Vidyanti, Indarwati Setyaningsih, Abdul Gofir, Cempaka Thursina Srie Setyaningrum, Christantie Effendy, Nurhuda Hendra Setyawan, Ismail Setyopranoto

**Affiliations:** 1 Department of Neurology, Faculty of Medicine, Public Health, and Nursing, Universitas Gadjah Mada, Yogyakarta, 55281 Indonesia; 2 Department of Neurology, Dr. Sardjito General Hospital, Yogyakarta, 55281 Indonesia; 3 Department of Medical-Surgical Nursing, Faculty of Medicine, Public Health, and Nursing, Universitas Gadjah Mada, Yogyakarta, 55281 Indonesia; 4 Department of Radiology, Faculty of Medicine, Public Health, and Nursing, Universitas Gadjah Mada, Yogyakarta, 55281 Indonesia

**Keywords:** Imaging marker, Medial temporal atrophy (MTA), Koedam score, Cognitive function, Dementia, Neuroscience, Neurology

## Abstract

Although medial temporal atrophy (MTA) and parietal atrophy (Koedam score) have been used to diagnose Alzheimer’s disease (AD), early detection of other dementia types remains elusive. The study aims to investigate the association between these brain imaging markers and cognitive function in dementia. This cross-sectional study collected data from the Memory Clinic of Dr. Sardjito General Hospital Yogyakarta, Indonesia from January 2020 until December 2022. The cut-off value of MTA and Koedam score was set with Receiver Operating Curve. Multivariate analysis was performed to investigate the association between MTA and Koedam score with cognitive function. Of 61 patients, 22.95% had probable AD, 59.01% vascular dementia, and 18.03% mixed dementia. Correlation test showed that MTA and Koedam score were negatively associated with Montreal Cognitive Assessment-Indonesian Version (MoCA-INA) score. MTA score ≥ 3 (AUC 0.69) and Koedam score ≥ 2 (AUC 0.67) were independently associated with higher risk of poor cognitive function (OR 13.54, 95% CI 1.77–103.43, *p* = 0.01 and OR 5.52, 95% CI 1.08–28.19, *p* = 0.04). Higher MTA and Koedam score indicate worse cognitive function in dementia. Future study is needed to delineate these findings as prognostic markers of dementia severity.

## Introduction

Brain atrophy, resulting from a variety of degenerative neuropathologies and vascular disorders, is known to be a precursor to dementia in later life^1,2^. Both temporal atrophy and posterior atrophy exhibit a robust correlation with the onset of dementia. The extent of atrophy is directly linked to the severity of cognitive impairment observed in dementia patients^3^. Moreover, the degree of cognitive decline in dementia patients is associated with increased morbidity and mortality rates^4,5^.

Within the temporal region, certain critical structures play a pivotal role in cognitive processes. Notably, the hippocampus and limbic system contribute significantly to cognitive functioning. Damage or a reduction in the volume of these structures due to degenerative processes, as well as compromised vascularization of the middle cerebral artery and its branches, are closely linked to abnormalities in visuospatial abilities, memory, executive function, processing speed, and language. Prior research has demonstrated that medial temporal atrophy is associated with the progression of Alzheimer’s dementia, with a Hazard Ratio (HR) of 1.68 (95% Confidence Interval (CI) 1.20–2.35)^3,6,7^.

Both temporal and posterior atrophy are implicated in cognitive impairment. Posterior atrophy, whether degenerative or stemming from abnormal vascular involvement of the posterior cerebral artery, encompasses the atrophy of key regions such as the parietal lobe, precuneus, posterior cingulate gyrus, and parieto-occipital sulcus^8,9^. The presence of posterior atrophy results in disturbances in visual and spatial abilities, executive function, as well as verbal fluency, working memory, and episodic memory. This is due to the intricate interplay between the posterior parietal and temporal areas in cognitive functioning^9^.

Currently, the diagnosis of dementia primarily relies on established diagnostic criteria and the outcomes of neuropsychological assessments^10,11^. However, the examination of affected brain areas is not commonly applied despite the potential variation in symptoms exhibited by patients. Diverse symptoms among patients necessitate tailored therapeutic approaches^12^. The Visual Rating Scale (VRS) is a volumetric assessment of the brain based on the results of CT (Computed Tomography) scans or Magnetic Resonance Imaging (MRI). The VRS enables the volumetric evaluation of several brain regions, particularly those associated with cognitive function^13^. Among various types of brain volumetric assessments, specific markers can be employed to identify brain atrophy linked to cognitive function. These markers include the Medial Temporal Atrophy (MTA) score for assessing temporal atrophy and the Koedam score for evaluating posterior parietal atrophy^13,14^.

Hence, it is essential to undertake research that substantiates the role of imaging markers such as the Medial Temporal Atrophy (MTA) score and the Koedam score in appraising cognitive function in individuals with any type of dementia. This study aims to investigate the association between MTA and Koedam score with cognitive function among any type of dementia.

## Methods

### Study design and participants

This research is a cross-sectional study using secondary data from medical records of dementia patients who visited the Memory Clinic at the Dr. Sardjito General Hospital Yogyakarta from January 2020 to December 2022. The inclusion criteria were: (1) Patients aged ≥ 45 years; (2) At least 3 years of education, and (3) Diagnosed with all types of dementia based on the diagnostic criteria^15^ (probable Alzheimer’s dementia, vascular, or mixed type) and brain MRI showing brain atrophy. Exclusion criteria in this study are (1) Structural lesions due to diseases of the central nervous system, namely tumors, trauma, surgery, and brain infections, (2) Patients suffering from other diseases that manifest in cognitive disorders, such as hepatitis and autoimmune, (3) Artifact in Brain MRI.

### Data collection

#### Demographic and clinical characteristics

The demographic data included age, sex, working status before sickness, and education. The clinical data included blood pressure, laboratory finding (albumin and lipid profile), existence of risk factors (hypertension, diabetes mellitus, dyslipidemia, stroke, and cardiovascular disease), MTA and Koedam score from brain MRI. We did not include the data related to dementia’s treatment because all patients receive similar standard treatment according to national guideline of dementia in Indonesia^16^.

#### Measurement of MTA and Koedam score

Volumetric brain MRIs were performed on all dementia patients in Memory Clinic by using the Philips 1.5 Tesla Multiva MRI machine (Philips HealthCare, Best, Netherlands) and the Siemens 3 Tesla Sykra (Siemens, Erlangen, Germany), employing isotropic 3D T1-weighted echo gradient sequences, FFE and MPRAGE, respectively. The slice thickness acquisition was set at 0.6 × 0.6 × 0.6 mm and was reconstructed to 0.9 × 0.9 × 0.9 mm for display on the Osirix MD version 8.0 workstation software (Pixmeo SARL). The Koedam and MTA scores were assessed by a single neuroradiologist (NHS) with over five years of clinical experience. The qualitative assessment of the MTA score was based on the degree of atrophy in the choroid fissure, temporal horn, and hippocampal volume from the coronal plane, as described by Frisoni et al.^17^. MTA 0 was characterized with normal choroid fissure, temporal horn, and hippocampal volume, MTA 1 was characterized with slight widened choroid fissure, MTA 2 was characterized with moderate widened choroid fissure, slight enlargement of temporal horn, and mild decrement of hippocampal volume, MTA 3 was characterized by markedly widened choroid fissure, moderate enlargement of temporal horn, and moderate decrement of hippocampal volume, and MTA 4 was characterized by markedly widened choroid fissure, markedly enlargement of temporal horn, and markedly decrement of hippocampal volume^18,19^.

The Koedam score’s qualitative assessment was assesed on the degree of parietal atrophy from the sagittal plane, as outlined by Koedam et al.^20^. Koedam score 0 showed normal sulci and no present atrophy of the precuneus while Koedam score 1, 2, and 3 showed mild, moderate, and severe widening of the sulci and atrophy of the precuneus, respectively^19^. Koedam score 0 indicated no cortical atrophy, Koedam score 1 was characterized with mild parietal cortical atrophy with slight widening of the posterior cingulate and parieto-occipital sulcus, Koedam score 2 showed moderate parietal cortical atrophy with significant widening of sulci, and Koedam score 3 was characterized with severe parietal atrophy (“knife blade” atrophy)^21^. MRIs with motion artifacts caused by patients’ movements during the examination were considered distortive and were consequently excluded from this study.

#### Cognitive assessment

We collected the data related to the cognitive function of dementia patients measured by the Montreal Cognitive Assessment-Indonesian Version (MoCA-INA). MoCA-INA is a 30-point cognitive screening tool that has been validated for the Indonesia population. It has been widely accepted and commonly used in the clinical and community setting in Indonesia^22,23^. We categorized the cognitive function as poor if MoCA score < 18 and better if MoCA ≥ 18 as reported by prior study^24^. The scoring of MoCA-INA is based on guidelines issued by Indonesia Neurological Association. The guideline includes how the test is administered and scored^25^.

### Statistical analysis

To analyze the statistical differences between variables, we used Kolmogorov–Smirnov test to determine the normality of numerical data, an independent t-test (for continuous variables), Mann–Whitney (for variables not normally distributed), and Chi-Square test (for categorical variables). The correlation between MTA and Koedam Score with MoCA-INA score were analyzed using Pearson Correlation Test. To determine the cut-off point of MTA and Koedam score, we performed Receiver Operating Characteristics (ROC) curve analysis and Youden Index. The crude odds ratios of MTA and Koedam Score associated with dementia patients’ cognitive function were measured using bivariate analysis with categorization from the cut-off derived by the ROC curve. Finally, multivariate logistic regression analysis was performed to measure the contribution of MTA and Koedam Score to the cognitive function. Model 1 tested the main effect (contribution) of MTA and Koedam score after controlling demographic variables (age and education). Model 2, in addition with controlling demographic variables, we also controlled variables such as dementia type and history of diseases (hypertension, cardiovascular disease, diabetes, dyslipidemia, and stroke). Model 3 we controlled all variables: demographic (Model 1), dementia type and history of disease (Model 2), and laboratory findings (albumin, cholesterol, triglyceride, HDL, LDL), thus the main effect of MTA and Koedam score were independently associated with poor cognitive function. All statistical analyses were assessed by SPSS software version 2.3 (IBM Co. Ltd, NY, USA). A p value of < 0.05 in two-tailed test indicated statistical significance.

### Ethics approval and consent to participate

All procedures performed in the present study were in accordance with ethical standards of the institutional research committees and with The 2013 Declaration of Helsinki. Ethical approval for this study was obtained from the Medical and Health Research Ethics Committee of the Faculty of Medicine, Public Health, and Nursing, Universitas Gadjah Mada, Yogyakarta, Indonesia (EC No. KE/FK/0288/EC). The anonymity of the participants was strictly maintained. Informed consent was obtained from all subjects and/or their legal guardian(s) for the use of their clinical data in this study.

## Results

### Demographic and clinical characteristics

Table 1 presents the baseline characteristics of dementia patients. Fourty-one patients had poor cognitive function and 20 patients had better cognitive function. The mean age of patients with poor and better cognitive function was 67.66 ± 10.61 years and 68.10 ± 8.14 years, respectively (*p* = 0.87). There were no differences in risk factors and laboratory findings for both groups.

**Table 1:** Baseline characteristics between the dementia patients with poor and better cognitive function.

	Poor cognitive function (MoCA-INA < 18) (n = 41)	Good cognitive function (Moca-Ina ≥ 18) (n = 20)	*p*	OR (95% CI)
Demographical data				
Sex				1.92 (0.58–6.32)
a. Female	16 (39.00%)	5 (25.00%)	0.28^€^	
b. Male	25 (61.00%)	15 (75.00%)		
Working status before sickness				1.29 (0.83–2.04)
a. Jobless	31 (75.61%)	12 (60.00%)	0.21^€^	
b. Work	10 (24.39%)	8 (40.00%)		
Age	67.66 ± 10.61	68.10 ± 8.14	0.87^	–
Education (years)	15.00 (6.00–23.00)	16.00 (6.00–29.00)	0.52^#^	–
Clinical characteristics				
Dementia type				–
a. Alzheimer’s dementia	9 (22.00%)	5 (25.00%)	0.90^€^	
b. Vascular dementia	24 (58.50%)	12 (60.00%)		
c. Mixed type dementia	8 (19.50%)	3 (15.00%)		
Cardiovascular disease				0.92 (0.61–1.36)
a. Yes	12 (29.27%)	7 (35.00%)	0.65^€^	
b. No	29 (70.73%)	13 (65.00%)		
Hypertension				1.82 (0.58–5.63)
a. Yes	30 (73.20%)	12 (60.00%)	0.29^€^	
b. No	11 (26.80%)	8 (40.00%)		
Diabetes mellitus				0.58 (0.19–1.70)
a. Yes	15 (36.60%)	10 (50.00%)	0.32^€^	
b. No	26 (63.40%)	10 (50.00%)		
Dyslipidemia				0.70 (0.24–2.07)
a. Yes	21 (51.20%)	12 (60.00%)	0.52^€^	
b. No	20 (48.80%)	8 (40.00%)		
Stroke				1.30 (0.42–4.06)
a. Yes	29 (70.70%)	13 (65.00%)	0.65^€^	
b. No	12 (29.30%)	7 (35.00%)		
Laboratory findings				
Albumin (g/dL)	4.08 ± 0.36	4.02 ± 0.62	0.63^	
Total cholesterol (mg/dL)	181.37 ± 47.71	193.40 ± 39.96	0.33^	
Triglyseride (mg/dL)	104.00 (3.00–344.00)	132.00 (23.50–449.00)	0.59^#^	
HDL (mg/dL)	47.00 (28.00–87.00)	45.00 (30.00–84.00)	0.90^#^	
LDL (mg/dL)	118.73 ± 35.19	123.95 ± 33.75	0.58^	

*MTA* medial temporal atrophy, *OR* odds ratio, *CI* confidence interval.

*p < 0.05.

^€^Chi-Square test.

^#^Mann Whitney test.

^Independent t-test.

### Correlation of MTA and Koedam score with dementia patients’ cognitive function

A higher MTA score correlates with a lower MoCA-INA score (r =  − 0.38, *p* < 0.05). This indicates that an increment in 1 point of the MTA score is associated with a corresponding decrease in the MoCA-INA score, up to a maximum decrease of 2.98 points (Table 2). Furthermore, a higher Koedam score also correlates with a lower MoCA-INA score (r =  − 0.28; *p* < 0.05). An increment in 1 point of Koedam score will cause a corresponding decrease in the MoCA-INA score, up to a maximum decrease of 2.81 points (Table 2).

**Table 2:** The correlation between the MTA and Koedam score with MoCA-INA score of dementia patients.

Score	Constant	B	*p*	R^2^
MTA	19.98	− 2.98	0.004	0.135
Koedam	18.39	− 2.81	0.027	0.080

*MTA* medial temporal atrophy, *CI* confidence interval.

*p < 0.05; Pearson correlation test.

### Categorization of MTA and Koedam score and dementia patients’ cognitive function

**Figure 1:**
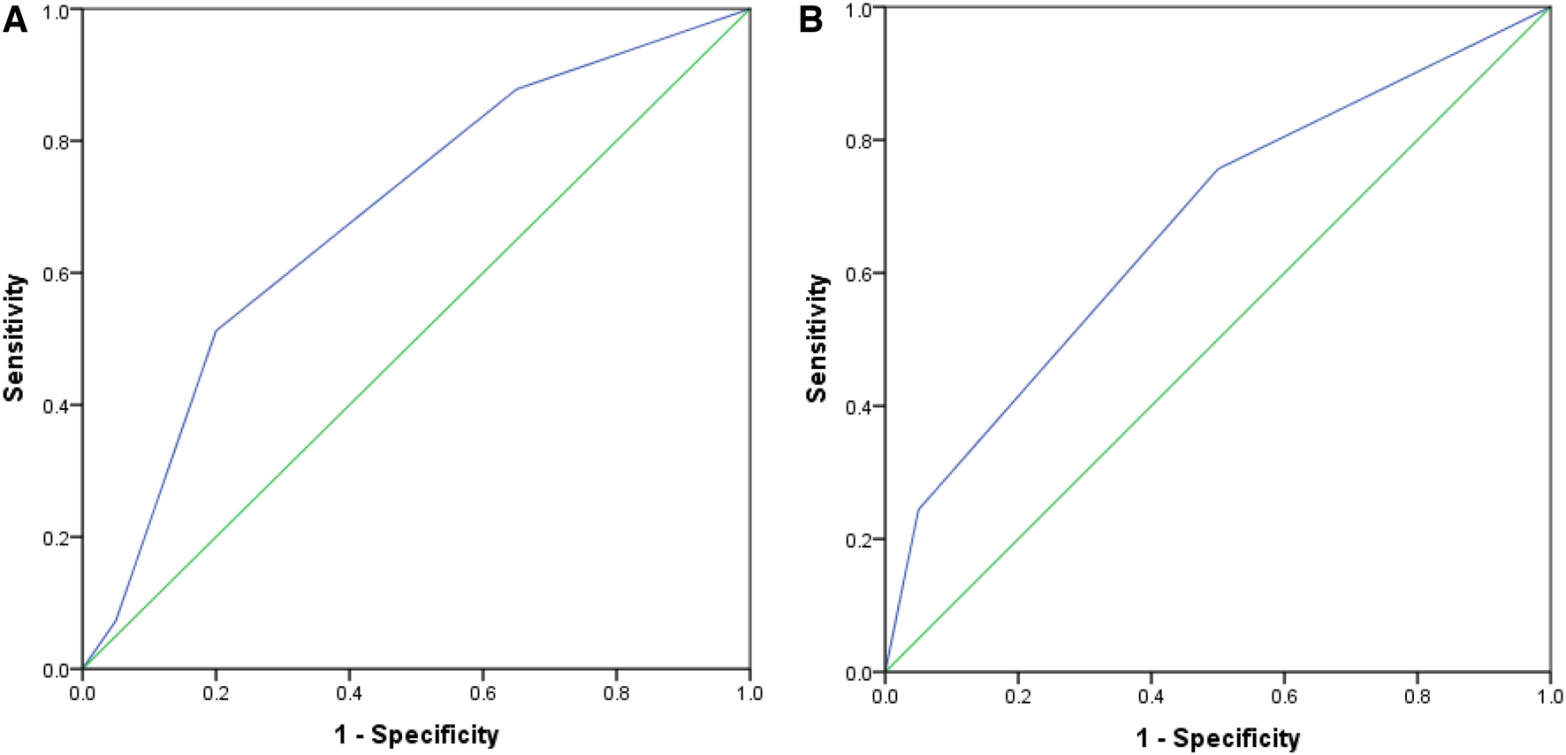
(**A**) ROC curve for the discrimination quality of MTA score in dementia patients (weak categorization). MTA score cut-off point of 3 with AUC 0.69 was used. (**B**) ROC curve for the discrimination quality of Koedam scores in dementia patients (weak categorization). Koedam score cut-off point of 2 with AUC 0.67 was used. (**A**) ROC curve for MTA score in dementia patients. (**B**)ROC curve for Koedam score in dementia patients.

To determine the cut-off point of MTA and Koedam score in discriminating dementia patients with poor and better cognitive function, we performed ROC analysis. We found that the cut-off point for MTA score was 3 with Area Under Curve (AUC) of 0.69 (Fig. 1A). Meanwhile, the cut off point for Koedam score was 2 with the AUC 0.67 (Fig. 1B). By using these cut-off points, we subsequently analyzed the association between MTA and Koedam Score with the cognitive functioning of dementia patients in bivariate and multivariate analysis.

Table 3 shows a bivariate analysis of the cognitive function with the categorization of MTA and Koedam Scores. Patients with higher MTA scores (≥ 3) were more likely to have poor cognitive function than those with lower MTA scores (OR 4.20, 95% CI 1.19–14.74, *p* = 0.02). Patients with higher Koedam scores (≥ 2) were more likely to have poor cognitive function than the counterpart group (< 2) (OR 3.01, 95% CI 1.01–9.59, *p* = 0.04). Then patients with a combination of higher MTA scores (≥ 3) and higher Koedam Scores (≥ 2) were more likely to have poor cognitive function than patients with low MTA scores (< 3) and low Koedam scores (< 2) (OR 11.33, 95% CI 1.86–69.08, *p* = 0.008).

**Table 3:** Bivariate analysis between MTA and Koedam score with the cognitive function.

	Poor cognitive function (MoCA-INA < 18) (n = 41)	Better cognitive function (Moca-Ina ≥ 18) (n = 20)	*p*	OR (95% CI)
MTA score				
a. ≥ 3	21 (51.22%)	4 (20.00%)	0.02^€^*	4.20 (1.19–14.74)
b. < 3	20 (48.78%)	16 (80.00%)	Ref	
Koedam score				
a. ≥ 2	31 (75.60%)	10 (50.00%)	0.04^€^*	3.01 (1.01–9.59)
b. < 2	10 (24.40%)	10 (50.00%)	Ref	
Combination				
a. MTA ≥ 3 and Koedam ≥ 2	17 (41.50%)	2 (10.00%)	0.008^€^*	11.33 (1.86–69.08)
b. MTA < 3 and Koedam < 2	6 (14.60%)	8 (40.00%)	Ref	

*MTA* medial temporal atrophy, *OR* odds ratio, *CI* confidence interval.

*p < 0.05.

^€^Chi-Square test.

### Association between MTA and Koedam scores with the cognitive function

To investigate whether MTA and Koedam score were independently associated with the cognitive function, we performed a multivariate logistic regression analysis. Table 4 present the multivariate logistic regression analysis of factors associated with poor cognitive function after adjustment by controlling multiple covariates. In Model I, we controlled the demographic characteristics (age and education). Patients with higher MTA (≥ 3) showed higher risk to have poor cognitive function (OR 4.12, 95% CI 1.09–15.48, *p* = 0.04).

**Table 4:** Multivariate logistic regression of factors associated with the poor cognitive function after adjustment with covariates.

Variable (risk vs reference)	OR	CI 95%	p
Model I			
MTA (≥ 3 vs < 3)	4.12	1.09–15.48	0.04*
Koedam (≥ 2 vs < 2)	2.97	0.89–9.92	0.08
Age (years)	0.98	0.92–1.05	0.55
Education (years)	0.93	0.82–1.06	0.31
Model II			
MTA (≥ 3 vs < 3)	11.91	1.45–97.92	0.02*
Koedam (≥ 2 vs < 2)	5.06	1.15–22.13	0.03*
Age (years)	0.96	0.89–1.05	0.42
Education (years)	0.91	0.79–1.06	0.25
Dementia type (Alzheimer disease)	0.22	0.02–2.54	0.23
Dementia type (dementia vascular)	0.61	0.08–4.54	0.64
Hypertension	0.20	0.03–1.36	0.10
Cardiovascular disease	4.17	0.85–20.64	0.08
Diabetes	0.37	0.09–1.54	0.17
Dyslipidemia	0.35	0.07–1.80	0.21
Stroke	0.75	0.12–4.52	0.75
Model III			
MTA (≥ 3 vs < 3)	26.96	2.07–351.79	0.01*
Koedam (≥ 2 vs < 2)	7.62	1.02–6.91	0.048*
Age (years)	0.98	0.89–1.08	0.74
Education (years)	0.90	0.76–1.07	0.25
Dementia type (Alzheimer disease)	0.14	0.01–2.35	0.17
Dementia type (dementia vascular)	0.35	0.04–3.32	0.37
Cardiovascular disease	0.08	0.01–0.89	0.04
Hypertension	4.87	0.81–29.34	0.08
Diabetes	0.22	0.04–1.31	0.09
Dyslipidemia	0.24	0.04–1.55	0.13
Stroke	0.46	0.05–3.94	0.48
Albumin	3.51	0.57–21.42	0.17
Cholesterol	0.97	0.91–1.03	0.27
Triglyceride	1.01	0.99–1.02	0.61
HDL	0.99	0.91–1.07	0.77
LDL	1.04	0.97–1.11	0.21

*MTA* medial temporal atrophy, *OR* odds ratio, *CI* confidence interval.

*p < 0.05.

For model II, we adjusted the odds ratio of MTA and Koedam score by controlling the demographical and clinical characteristics. We found that patients with higher MTA (≥ 3) and Koedam (≥ 2) scores were likely to have poor cognitive function (OR 11.91, 95% CI 1.45–97.72, *p* = 0.02; OR 5.06, 95% CI 1.15–22.13, *p* = 0.03).

For model III, we adjusted the odds ratio of MTA and Koedam score by controlling all variables (the demographical, clinical characteristics, and laboratory findings). We found that patients with higher MTA (≥ 3) and Koedam (≥ 2) scores were likely to have poor cognitive function (OR 26.96, 95% CI 2.07–351.79, *p* = 0.01; OR 7.62, 95% CI 1.02–6.91, *p* = 0.048).

## Discussion

In the present study, we demonstrated that dementia patients with higher MTA (≥ 3) and Koedam (≥ 2) score were more likely to have a poor cognitive function. This finding is applicable to various types of dementia, encompassing not only Alzheimer’s disease but also vascular and mixed dementia.

The findings in the present study corroborates previous study that showed a relationship between the results of the VRS examination and the severity of cognitive function in dementia patients^13^. A higher MTA score is significantly correlated with lower score of Mini Mental State Examination (MMSE) of dementia patients (p = 0.00095) and a higher Koedam score is associated with a worse Clinical Dementia Rating (CDR) score (p < 0.05)^13,14^. Nonetheless, the conclusions drawn in previous studies were limited in scope, as they exclusively pertained to patients with Alzheimer’s dementia.

The Medial Temporal Lobe Atrophy (MTA) score evaluates hippocampal atrophy on a five-point scale, where a higher score reflects a greater degree of atrophy. Previous studies have shown that the MTA score is associated with memory impairment in patients with Alzheimer’s dementia and the amnestic type of Mild Cognitive Impairment (MCI). MTA is correlated significantly with the memory domain or memory recall with R^2^ = 0.100 (p = 0.043)^26,27^.

The MTA score has a sensitivity of 85% and a specificity of 82% in diagnosing patients with Alzheimer’s Dementia. A higher MTA score will be associated with a lower MMSE score (B =  − 0.2; p < 0.01)^20^. In the present study, the relationship between a high MTA score with poor cognitive among dementia patients, including patients with vascular dementia (VaD), may indicate a pre-existing Alzheimer’s pathology in those with VaD. Furthermore, knowing the MTA score could also assist clinicians in predicting the progression of dementia.

Some crucial structures in the temporal region are responsible for cognitive processes. The hippocampus and limbic system are responsible for cognitive function. The hippocampus forms declarative memory. The hippocampus is connected to the presubiculum structure which is located posterosuperior to the parahippocampal gyrus^28^. This presubiculum will connect with the cingulum, precuneus, occipital lobe, entorhinal cortex, and perirhinal cortex that processes spatial information. The entorhinal cortex connects the temporal lobe with the frontal, temporal, parietal, and hippocampus lobes. Meanwhile, the underlying white matter is responsible for memory consolidation and rapid encoding in the formation of new associations^28^. Damage or decline in the volume of these structures results from degenerative processes and disturbances in the vascularization of the middle cerebral arteries and their branches closely related to abnormalities in the visuospatial domain, memory, executive function, processing speed, and language. Previous studies have shown that medial temporal atrophy is associated with the progression of Alzheimer’s dementia with a Hazard Ratio (HR) of 1.68 (95% CI 1.20–2.35)^3,6,7^.

In addition to the role of MTA score, previous studies have also shown that posterior atrophy was associated with dementia progression with an HR of 2.24 (95% CI 1.49–3.36)^3^. Posterior atrophy, measured by Koedam score, is a potential biomarker in the early onset of Alzheimer’s dementia and atypical Alzheimer’s dementia including posterior cortical atrophy (PCA)^20^. Examination of the Koedam score is very helpful for clinicians in diagnosing dementia, particularly in patients with atypical symptoms. It also helps differentiate the types of dementia with a sensitivity of 57% and a specificity of 95%. Moreover, prior findings revealed that the Koedam score is associated with a lower working memory score (R^2^ = 0.106; p = 0.043) and MMSE score (B =  − 1.8, p < 0.01)^20,26,27^. Similar with MTA score, most studies investigating the role of the Koedam score in aiding the diagnosis of dementia have primarily focused on patients with Alzheimer’s disease. However, research focusing on the relationship between Koedam score and cognitive function in VaD patients is still scarce. In the present study, a higher Koedam score corresponds with poor cognitive functioning in all type of dementia, including VaD, may also indicate a pre-existing Alzheimer’s pathology.

Posterior atrophy, either due to degenerative or vascular abnormalities of the posterior cerebral arteries, includes atrophy of the parietal lobe, precuneus, posterior cingulate gyrus, and parieto-occipital sulcus^8,9^. Posterior atrophy will manifest in abnormalities in visual, spatial, and executive function, verbal fluency, working memory as well as episodic memory because of the role and connection between the posterior parietal and temporal regions in cognitive function^9^. This cognitive function disorder is associated with the connection between the Dorsal Attention Network and the frontoparietal network, which includes the superior and inferior parietal lobules and the intraparietal sulcus^9^.

This study was similar to previous study, which MTA and Koedam score was negatively correlated with MMSE score. Previous study reported that MTA score had strong negative correlation with MMSE score in AD patients (p = 0.000951)^13^. This condition reflected the pathological condition in AD patients which was dominated by atrophy in hippocampus and responsible to cognitive impairment (R =  − 0.7827, *p* = 0.000951). Additionally, Koedam score was also negatively correlated with MMSE score in AD and VaD patients (R =  − 0.1425, *p* = 0.628) although it was not statistically significant because the posterior atrophy is attributed to noncognitive symptoms in dementia^13^.

Moreover, the cut-off points of AUC in the present study corroborates the previous study. The previous study showed that the AUC for discriminating mild cognitive impairment from normal control was 0.589 for MTA and 0.585 for Posterior atrophy (Koedam score)^29^. However, the AUC is higher in another study conducted by Ferreira et al.^30^. They reported AUC 0.838 for MTA and 0.567 for Koedam score, in which they used cut-off point score of ≥ 1 for Koedam score for all age, and different cut-off points for MTA for different age range^30^. The different findings in the present study may be due to different methodology in which the cut-off point in the present study was to discriminate between good and poor cognitive functioning among dementia patients, not for discriminating between normal control and dementia. Furthermore, this could also be due to relatively small sample size, varied background of dementia type, and varied range of patient’s age. Nevertheless, the AUC of MTA and Koedam score in the present study still have moderate accuracy to predict the cognitive functioning of all type of dementia patients in Indonesia. However, it has been established that dementia patients with older age would have higher score of MTA^30,31^. Therefore, further study with larger sample size is needed to yield stronger AUC for general population and should be adjusted with different age range.

Although all studies demonstrated that MTA score was associated with cognitive function in dementia patients, some studies revealed a contradictory finding related to Koedam score^13,20^. Previous studies showed that the Koedam score was not correlated with cognitive function. It is a complementary measure instead, which is more related to non-cognitive symptoms in dementia (such as agitation and aggression)^32–34^. Different patterns of atrophy will also lead to different symptoms in AD patients and the posterior atrophy may still be beneficial to discriminate AD from FTD^27,35,36^. This discrepancy could also be attributed to variations in methodologies employed, which may yield divergent results. Furthermore, when considering vascular dementia, cognitive outcomes are more likely to be influenced not solely by brain atrophy but, more significantly, by factors such as the cerebral hemodynamic, size and location of infarcts, along with the presence of other vascular risk factors^37–39^.

### Limitations of the study

This study has several limitations. First, the result could not be generalized to all population due to relatively small sample size. Second, we did not analyze the effect of medication in this study because almost all participants received similar standard treatment for dementia. In addition, we did not analyze the relationship between each domain of the cognitive function in MoCA with the MTA and Koedam score. Finally, this present study exclusively relied on cross-sectional data, preventing the establishment of a causal link between MTA and Koedam score with cognitive impairment. However, the observed direction of influence was consistent with prior studies.

## Conclusion

In conclusion, this study confirmed that higher MTA and Koedam scores are associated with poor cognitive functioning in dementia patients, particularly Alzheimer’s, vascular, and mixed dementia. These findings may contribute to assist clinicians to predict the level of cognitive function among dementia patients, thus prompt intervention could be made to prevent the development of more severe dementia.

## Data Availability

Dataset generated during and/or analysed during the current study are available in the zenodo repository, https://zenodo.org/records/10445663.

## Abbreviations

AUC: Area under curve
CDR: Clinical dementia rating
CT: Computed tomography
HR: Hazard ratio
MCI: Mild cognitive impairment
MMSE: Mini Mental State Examination
MoCA: Montreal cognitive assessment
MoCA-INA: Montreal cognitive assessment-Indonesian version
MRI: Magnetic resonance imaging
MTA: Medial temporal atrophy
ROC: Receiver operating characteristics
VRS: Visual rating scale
